# Polycystic ovarian syndrome awareness among females in the UAE: a cross-sectional study

**DOI:** 10.1186/s12905-023-02318-y

**Published:** 2023-04-17

**Authors:** Balkis Zaitoun, Abdullah Al Kubaisi, Noora AlQattan, Yahya Alassouli, Alshaima Mohammad, Huriya Alameeri, Ghada Mohammed

**Affiliations:** 1 College of Medicine, University of Sharjah, Sharjah, United Arab Emirates; 2 Clinical Sciences Department, College of Medicine, University of Sharjah, Sharjah, United Arab Emirates

**Keywords:** Polycystic ovarian syndrome, Polycystic ovary syndrome, Stein-leventhal syndrome, Hyperandrogenism, Ovarian diseases

## Abstract

**Background:**

Polycystic Ovarian Syndrome (PCOS) is a common hormonal disorder affecting females of reproductive age. Clinical guidelines recommend following the diagnostic criteria of PCOS based on an appropriate assessment of the patient’s clinical presentation. Sufficient awareness among the population will prompt females to seek medical attention when necessary. This study aimed to assess knowledge and awareness about PCOS among females above the age of 18 years in the United Arab Emirates (UAE) and to correlate the level of awareness with women's backgrounds, demographics, and education levels.

**Methods:**

This was a cross-sectional study conducted in early 2020. 430 females over the age of 18 years were conveniently selected and interviewed in the UAE using a 21-item questionnaire that assessed participants' awareness of PCOS as a term, its causes, symptoms, complications, treatment, and prevention. 414 entries were eligible for data analysis. IBM® SPSS® Statistics version 25 was used for data entry and analysis.

**Results:**

349 participants (84.3%) were familiar with the term PCOS. However, only 21.7% of them had sufficient awareness of the syndrome (95% CI = 17.77–25.71%). Being knowledgeable of PCOS was significantly associated with having a previous diagnosis (*p* = 0.002) and with studying or working in a medical field (*p* < 0.001). In addition, females who knew someone diagnosed with PCOS were 5 times more likely to be more aware compared to those who didn’t (95% CI = 2.5–10.8; *p* < 0.001). Age, education level, and nationality showed no correlation with the level of awareness.

**Conclusions:**

Overall, the level of PCOS awareness was insufficient in the study sample. Participants whose source of information was medical practitioners demonstrated more accurate knowledge. Accordingly, awareness of PCOS needs to be raised among females in the UAE, aiding early diagnosis and improving patient-oriented outcomes.

**Supplementary Information:**

The online version contains supplementary material available at 10.1186/s12905-023-02318-y.

## Background

Polycystic ovarian syndrome (PCOS) is one of the most common endocrinopathies affecting females of reproductive age with an estimated global prevalence of 4–20% [1]. The wide variation in prevalence rates across the world is attributed to the lack of unified diagnostic criteria among prevalence studies [1]. Moreover, PCOS is known to be significantly associated with obesity and metabolic syndrome [2]. Despite the evidence of the high prevalence of obesity and metabolic syndrome in the United Arab Emirates (UAE) [3, 4], there is limited data on the prevalence of PCOS in the same region.

The Endocrine Society guidelines recommend the use of Rotterdam criteria to diagnose females with PCOS. According to these criteria, a diagnosis of PCOS is confirmed by the presence of at least two out of the three following criteria: hyperandrogenism, ovulatory dysfunction (oligo- or anovulation), and morphologically polycystic ovaries. Hyperandrogenism and ovulatory dysfunction both manifest clinically, while polycystic ovaries can be detected on ultrasound assessment.[5, 6] Thus, the clinical picture is an essential pillar of PCOS diagnosis, which emphasizes the importance of awareness and knowledge about the symptoms and signs of PCOS.

PCOS clinical presentation may not be alarming to most women. In young adolescents, symptoms like weight gain, acne, menstrual irregularities, and abnormal hair distribution can be easily mistaken for common pubertal issues [7]. Thus, the disease can be overlooked until severe complications arise, most commonly infertility, the point at which most patients seek medical attention secondary to difficulty conceiving [8]. Furthermore, PCOS was linked to a higher risk of having endometrial cancer, type 2 diabetes mellitus, and cardiovascular diseases [9]. In other age groups like perimenopausal women, menstrual irregularities and the expected weight gain may mask the symptoms of PCOS among those women [10]. Consequently, there lies a common pattern of PCOS patients being under-evaluated and misdiagnosed [11, 12].

It has been evident that an early diagnosed PCOS case is easily managed compared to the more complex management of the later stages [13]. Nevertheless, awareness was found to be lacking in both healthcare professionals and females of the general population [8].

Multiethnic studies on PCOS have found a particularly higher prevalence of the syndrome among females of Mediterranean ethnicity [14]. Despite that, an extensive literature review revealed that only a few articles were found to be investigating PCOS prevalence in the Middle East and North Africa (MENA) region. There is an evident gap in the currently available literature. Articles addressing PCOS prevalence in the region are scarce and outdated. Moreover, limited and small sample sizes, non-probability sampling methods, and vague diagnostic criteria were used in these studies. Among the studies found, one study investigated the region-wide prevalence of PCOS in the MENA region, reporting a point prevalence of 2079.7 per 100,000 in 2019 [15]. Of particular note, a few papers measured the local prevalence in Qatar [16], Oman [17], Egypt [18, 19], Iran [20], and Syria[21]. For the Gulf region, a total of 7 articles in the Gulf Council Countries (GCC) are found addressing PCOS prevalence in the literature as of 2022 [16, 17, 22–26], one of which was conducted in Ras Al Khaimah (RAK), UAE [26].

There are only a few available studies evaluating PCOS awareness in the MENA region, either as a primary or a secondary objective [27–31]. Overall, awareness was found to be significantly low [27, 29, 30, 32]. Of interest, most studies showed that females were least aware of PCOS complications. This raises concerns as fear of irreversible complications could be the main drive for patients to seek early medical help.

In the UAE, a recent study assessed Emirati university students’ knowledge about reproductive health and PCOS, along with the prevalence of reproductive symptoms among these students. It was found that there is a high prevalence of symptoms in addition to major gaps in the participants’ knowledge [32]. An interventional study conducted in Ras Al Khaimah (RAK), UAE, assessed the effect of a structured, educational program about PCOS on university students’ knowledge. They found a statistically significant improvement in the knowledge post-intervention compared to the baseline [33].

Considering the gaps discussed in the literature, it was of interest to conduct this study to address the level of awareness of PCOS among females in the UAE, highlighting factors that might contribute to their knowledge and understanding of the syndrome.

## Methods

This cross-sectional study included females living in the UAE (nationals and non-nationals) aged 18 and above who speak English or Arabic, the two most widely spoken languages in the UAE. Any visiting females who were not UAE residents were excluded from the study sample.

A self-constructed questionnaire was used to assess participants’ knowledge about PCOS, based on data retrieved from Medscape [34], and in reference to other questionnaires used in similar studies with modifications tailored to the cultural background of the study population. It has a total of 21 close-ended questions divided into 4 main sections: Participant demographics (7 items), previous experience (2 items), knowledge about PCOS (11 items), and source of information (1 item).

The questionnaire begins with questions on the participant’s demographics, followed by a question on whether or not the participant is familiar with the term Polycystic Ovarian Syndrome and/or “PCOS”. In the recruitment process, participants who never heard of the term were asked to end the interview. Under the section on previous experience, participants were asked if they were previously diagnosed with the syndrome and if they knew someone with a diagnosis of PCOS. Participants were asked if they consider themselves aware of the different PCOS aspects, including symptoms, causes, prevention, complications, and treatment modalities. Those who considered themselves aware of any of the aspects were further assessed for the accuracy of their knowledge. Multiple mixed relevant and irrelevant options were provided, with “yes” / “no” / “I’m not sure” choices for each option. By the end of the questionnaire, participants provided their source of information by ticking all that applies from family and friends, media, medical professionals, and campaigns. The questionnaire was made available in two languages, English and Arabic [see Additional File: English and Arabic PCOS Study Questionnaires.pdf]. Participants had the choice to choose one according to their preference. The questionnaire was pilot-tested to approximate the time required to fill the questionnaire, which was found to be 5–6 min.

The sample size was calculated using the equation $n=\frac{4p(1-p)}{{ME}^{2}}$, in which *n* = sample size, *p* = prevalence, and ME = marginal error. The prevalence was assumed to be 50%, according to the WHO guidelines [35], since there was no similar study conducted in the UAE. Considering a maximum marginal error of 5%, an adequate number of respondents was estimated to be 400. Eventually, a total of 430 participants were recruited into the study.

Data were collected during January and February of 2020. A non-probability convenience sampling method was used to easily select female participants from the Sharjah population such as students’ dorms, libraries, malls, gyms, and supermarkets. Participants were interviewed by the female members of the research team to respect the local cultural norms since PCOS is a sex-based disease affecting females only. Participants were handed an information sheet clearly stating that completion of the questionnaire signifies obtained consent and that their voluntary participation is completely anonymous and carries no risk, with the right to withdraw at any stage.

### Data analysis

IBM® SPSS® Statistics for Macintosh, Version 25.0 was used to enter, clean, and analyze the data collected. 16 entries were excluded due to missing and non-applicable values, and the remaining 414 participants were analyzed. In addition, age and nationality variables were regrouped due to the wide variety of responses. For awareness assessment, a score was calculated for each participant based on their choice of answers, with those scoring higher than 60% being considered to be aware. In bivariate data analysis, awareness status was correlated with the variables of interest. Given the fact that all variables are categorical, the non-parametric chi-square test was used to obtain the p-value of each correlation. A p-value of 0.05 or less entails a significant correlation between the awareness status and the variable being tested.

### Ethical considerations

Ethical approval was granted by the Medical Research Ethics Committee of the College of Medicine and Health Sciences at the University of Sharjah (Ref. No. REC-20–02-04–03-S).

## Results

### A. Demographics

Of the 430 participants who were recruited into the study, 414 (*n*=414) were eligible for data analysis. Most participants fell under the age group 18-24 years. 57.7% of females enrolled in the study were non-local compared to 42.3% locals. Moreover, around 75% of the participants were single. The majority of enrolled participants were students, while 67.80% reported having a diploma/bachelor’s degree at the time of data collection. A scarce number of participants reported not entering a school at all (Table 1).

**Table 1:** Participants Demographics

Basic Demographic Data		Frequency (%)
Total number of participants: 414		
Age group:	18–24 years old	69.60%
	25–44 years old	22.20%
	45 + years old	8.20%
Nationality:	Emirati	42.27%
	Non-Emirati	57.73%
Marital Status:	Single	75.60%
	Married	20.77%
	Divorced	1.93%
	Widowed	1.69%
Educational level:	Didn’t go to School	0.73%
	Primary School	1.45%
	High School Diploma	23.73%
	Bachelor’s Degree/Diploma	67.80%
	Higher Studies	6.30%
Field of work/study:	Medical	47.30%
	Non-medical	52.70%
Working Status:	Student	66.40%
	Employed/Retired	25.60%
	Unemployed	8.00%

### B. Univariate analysis

#### Knowledge of PCOS and its aspects

As shown in Table 2, 84.30% of respondents were familiar with the term PCOS. Moreover, 59.90% stated knowing someone with a diagnosis of PCOS, while 18.60% were PCOS patients themselves.

**Table 2:** Participants’ Responses on PCOS Knowledge

**PCOS Knowledge and History**		**Frequency (%)**
Do you know the term PCOS?	Yes	84.30%
	No	15.70%
Have you ever been diagnosed with PCOS?	Yes	18.60%
	No	81.40%
Do you know anyone who has been previously diagnosed with PCOS?	Yes	59.90%
	No	40.10%

**Fig. 1:**
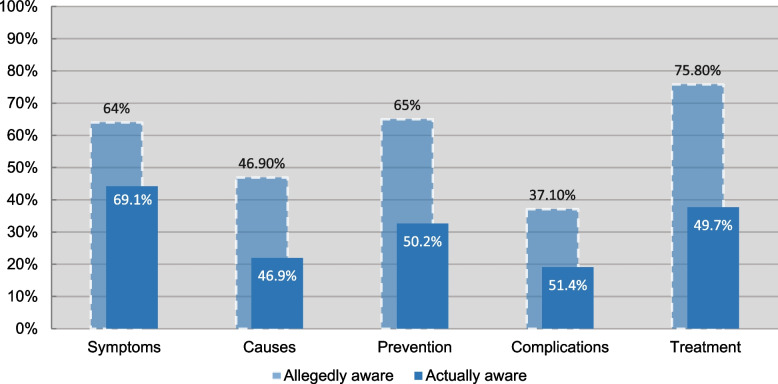
Participants Knowledge on Each Aspect of PCOS

Participants’ perceived knowledge was markedly inflated in comparison to the accurate post-assessment knowledge, as shown in Figure 1 which further demonstrates the knowledge gap related to each aspect.

#### Accuracy of participants responses

**Fig. 2:**
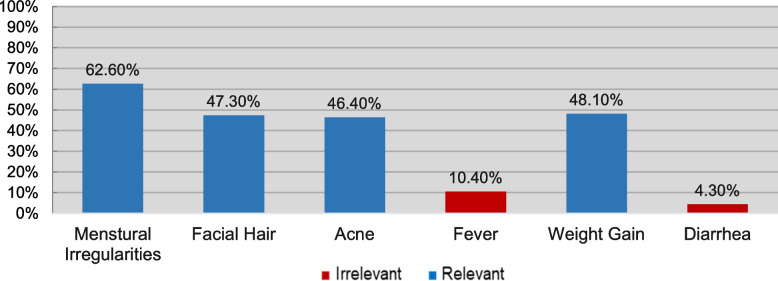
How Frequently was Each Symptom Chosen?

**Fig. 3:**
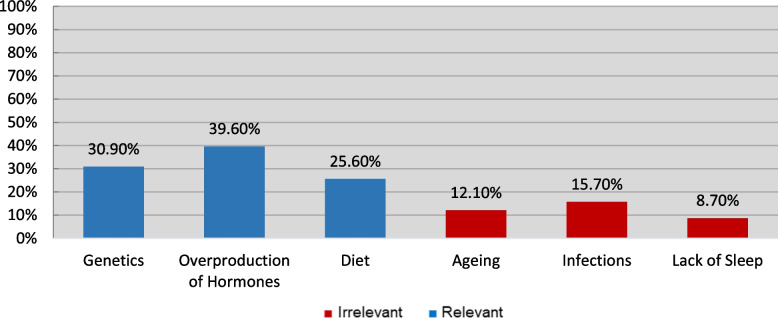
How Frequently was Each Cause Chosen?

**Fig. 4:**
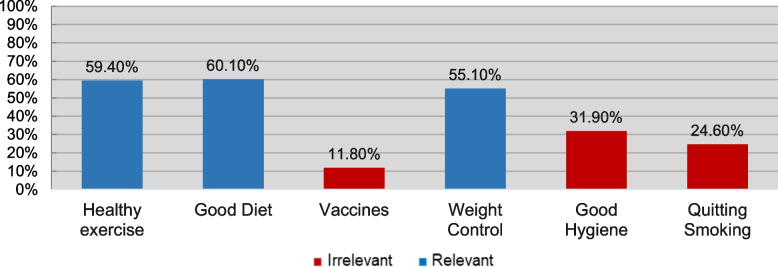
How Frequently was Each Preventive Method Chosen?

**Fig. 5:**
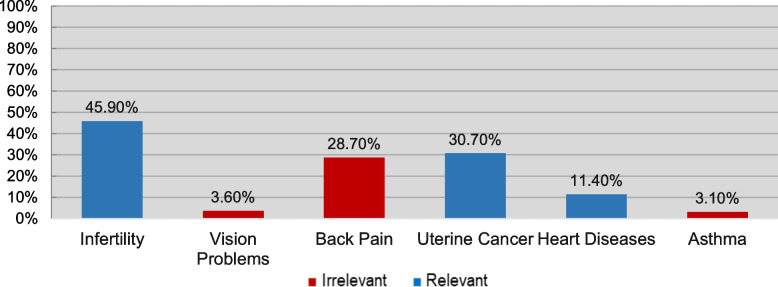
How Frequently was Each Complication Chosen?

**Fig. 6:**
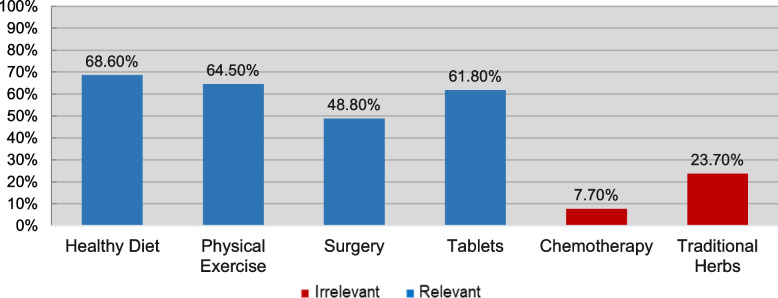
How Frequently was Each Treatment Method Chosen?

Figures 2, 3, 4, 5 and 6 demonstrate the frequency at which participants chose each item under every subsection. For the sake of clarity, columns of the relevant choices are shown in blue, and irrelevant ones in red. As demonstrated in Figure 2, the most frequently chosen PCOS symptom was menstrual irregularities, followed by weight gain and signs of hyperandrogenism (facial hair and acne).

Figure 3 illustrates participants’ response in terms of PCOS causes. As shown, overproduction of hormones was believed to be a cause by most, followed by genetics and then diet. A minority of participants falsely believed that infections, aging, and lack of sleep can cause PCOS.

Moreover, Figure 4 sums participants’ beliefs on PCOS preventive methods, which shows that the three most commonly chosen choices were ones that have been proven helpful in reducing the likelihood of getting PCOS. On the other hand, one-third and one-quarter of participants incorrectly believed that good hygiene and quitting smoking prevent PCOS, respectively. For PCOS complications, around half (51.40%) of enrolled participants considered themselves aware. Of those, around one-third (37.10%) demonstrated sufficient knowledge about the complications.

As Figure 5 demonstrates, infertility and uterine cancer were the most frequently chosen complications, followed by back pain, which is not a complication of PCOS. Interestingly, 88.60% of participants were not aware of the fact that PCOS can lead to heart disease.

Most participants agreed that a healthy diet and physical exercise can help manage PCOS, while traditional herbs and chemotherapy were the least frequently chosen options (Figure 6).

Furthermore, post-assessment analysis (Table 3) revealed that signs and symptoms (PCOS clinical presentation) was the aspect that people were most aware of, followed by treatment modalities, prevention, causes, and lastly, complications. All in all, 78.26% of participants fell under the non-aware group, compared to only 21.74% (95% CI = 17.77, 25.71) who demonstrated sufficient awareness.

**Table 3:** Participants Awareness Levels Post-assessment

PCOS Awareness	Frequency (%)
**Overall awareness**	**21.74%**
Signs and symptoms awareness	44.22%
Causes awareness	21.99%
Prevention awareness	32.63%
Complications awareness	19.06%
Treatment awareness	37.67%

### C. Source of information

**Fig. 7:**
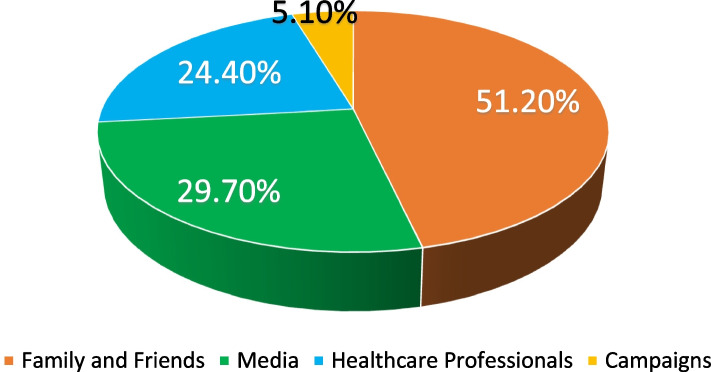
How Frequently was Each Source of Information Chosen?

**Fig. 8:**
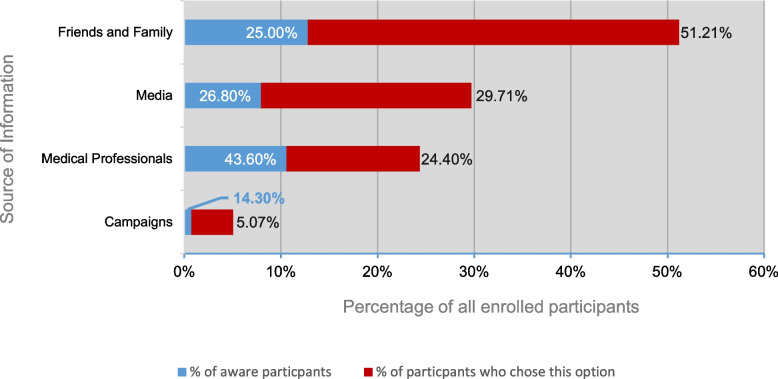
How Aware were Participants in Each Source of Information Group?

Figure 7 demonstrates how frequently was each source of information chosen. As interpreted, friends and family members were the most frequently chosen source, followed by media and healthcare professionals. Campaigns were chosen the least as a source of knowledge, accounting for only 5.10%. To demonstrate the awareness level per each group, Figure 8 shows the same percentages reported in Figure 7 but in red, with the addition of a blue bar that represents the percentage of those aware in each group.

### D. Correlation of PCOS awareness to the variables of interest

A statistically significant correlation was found between being aware of PCOS and the following variables: working/studying in a medical field, knowing someone with a PCOS diagnosis, having a previous diagnosis of PCOS, and reporting the source of information to be from a medical professional.

As table 4 illustrates, 33.5% of participants who knew someone with a diagnosis were aware of the syndrome compared to only 6.5% of those who didn’t (*p*<0.001).

**Table 4:** Cross-tabulation of the Relationship between Awareness and Knowing Someone with PCOS

			Awareness		Total
			Not Aware	Aware	
“Do you know anyone with PCOS?” responses	Yes	Count	165	83	248
		% within participants who know someone with a PCOS diagnosis	66.5%	33.5%	100.0%
		% within awareness group	62.0%	92.2%	69.7%
		% of Total	46.3%	23.3%	69.7%
	No	Count	101	7	108
		% within participants who do not know anyone with a PCOS diagnosis	93.5%	6.5%	100.0%
		% within awareness group	38.0%	7.8%	30.3%
		% of Total	28.4%	2.0%	30.3%
Total		Count	266	90	356
		% within awareness group	100.0%	100.0%	100.0%
		% of Total	74.7%	25.3%	100.0%

Reporting the source of information to be a medical professional was significantly linked to more awareness among participants (*p* = 0.000, Table 5). For reference, Fig. 8 demonstrates the awareness level per each source of information group.

**Table 5:** Cross-tabulation of the Relationship between Awareness and Choosing Medical Professionals as a Source of Information

			Awareness		Total
			Not aware	Aware	
Were medical professionals listed as one of the participant’s sources of information?	Yes	Count	57	44	101
		% within participants who chose medical professionals	56.4%	43.6%	100.0%
		% within awareness group	24.5%	50.6%	31.6%
		% of total	17.8%	13.8%	31.6%
	No	Count	176	43	219
		% within participants who did not choose medical professionals	80.4%	19.6%	100.0%
		% within awareness group	75.5%	49.4%	68.4%
		% of total	55.0%	13.4%	68.4%
Total		Count	233	87	320
		% within awareness group	100.0%	100.0%	100.0%
		% of total	72.8%	27.2%	100.0%

Moreover, participants working/studying in a medical field were twice more likely to be well-aware compared to those in a non-medical field (*p* < 0.001), refer to Table 6.

**Table 6:** Cross-tabulation of the Relationship between Awareness and Field of Work/Study

			Awareness		Total
			Not Aware	Aware	
Field of Work/Study?	Medical	Count	141	55	196
		% within participants in a medical field	71.9%	28.1%	100.0%
		% within awareness group	46.5%	69.6%	51.3%
		% of total	36.9%	14.4%	51.3%
	Non-medical	Count	162	24	186
		% within participants in a non-medical field	87.1%	12.9%	100.0%
		% within awareness group	53.5%	30.4%	48.7%
		% of total	42.4%	6.3%	48.7%
Total		Count	303	79	382
		% within awareness group	100.0%	100.0%	100.0%
		% of total	79.3%	20.7%	100.0%

Additionally, a higher percentage of previously diagnosed participants (39%) demonstrated sufficient awareness compared to only 21.6% among those without a previous PCOS diagnosis (*p* < 0.05) as shown in Table 7.

**Table 7:** Cross-tabulation of the Relationship between Awareness and Having a Previous Diagnosis of PCOS

			Awareness		Total
			Not aware	Aware	
“Have you been diagnosed?” responses	Yes	Count	47	30	77
		% within participants previously diagnosed with PCOS	61.0%	39.0%	100.0%
		% within awareness group	17.7%	33.3%	21.7%
		% of total	13.2%	8.5%	21.7%
	No	Count	218	60	278
		% within participants not previously diagnosed with PCOS	78.4%	21.6%	100.0%
		% within awareness group	82.3%	66.7%	78.3%
		% of total	61.4%	16.9%	78.3%
Total		Count	265	90	355
		% within awareness group	100.0%	100.0%	100.0%
		% of total	74.6%	25.4%	100.0%

As shown in Table 8, out of the 4 variables discussed above, knowing someone with PCOS had the most likelihood of being aware (5.16 times). On the other hand, participants with a previous PCOS diagnosis were 1.8 times as likely to be aware as participants without a previous diagnosis.

**Table 8:** Risk Estimate

	Knowing Someone with PCOS	Choosing Medical Professionals as a Source of Information	Medical field of work/study	Having a Previous Diagnosis
For cohort Awareness = Aware	5.164	2.219	2.175	1.805
For cohort Awareness = Not aware	.711	.702	.826	.778
N of Valid Cases	356	320	382	355

Lastly, no significant correlation was found between PCOS awareness and the following variables: educational level, age, nationality, and marital status.

## Discussion

This study aimed at evaluating the knowledge and awareness of Polycystic Ovarian Syndrome (PCOS) among the female population in the United Arab Emirates (UAE). Unfortunately, there was an evident lack of awareness among the study sample, remarkably of the syndrome’s complications. This was similarly reported in a previous study in the region [27]. Moreover, there was a statistically significant association between being sufficiently aware of the syndrome and the following: having a previous diagnosis, knowing someone with the syndrome, being in the medical field, and acquiring information from a medical professional. Nevertheless, participants mostly acquired their knowledge about the syndrome from family and friends, while medical professionals and awareness campaigns came last. This reflects the need to further promote awareness about PCOS and women’s health among the general population.

PCOS awareness was found to be 21.74%, which is relatively low despite the fact that a majority of participants (74.1%) achieved higher education, in addition to nearly half of them working or studying in the medical field. This finding was in line with similar studies in the region [27, 29, 30, 32]. Of all PCOS aspects assessed in this study (Table 3), signs and symptoms had the highest percentage of awareness (44.22%). On the other hand, complications had the least (19.06%), a finding that was similarly reported in other studies.[27, 31] For instance, a vast majority (88.60%) were not aware that PCOS increases the risk for heart disease, while almost a third of participants falsely believed that back pain is a complication of PCOS. Among PCOS complications, infertility is usually the one of most concern, as it can cause stress and psychological issues, further exacerbating patients’ quality of life [36]. Nevertheless, more than half were unaware of it as a complication of PCOS. Since a majority only seek medical attention when complications arise [8], this signifies that being aware of the syndrome’s signs and symptoms is not alarming enough for potential patients to seek the care they need, and fear of complications is probably the driving force. Such misconceptions can further extend the time to diagnosis, which has been linked to a higher rate of patient dissatisfaction [11] and a longer period of untreated disease course. This further increases the risk of complications, including infertility, psychological distress, and cardiovascular events [37].

The study findings suggest that awareness of the syndrome is strongly associated with being directly or indirectly affected by PCOS, as knowledge among participants was highly dependent on previous experience. Around two-thirds of aware participants already knew someone with a diagnosis, while one-fifth had a previous diagnosis of PCOS. It has been previously reported in one study that more than half of PCOS patients considered themselves to be “very aware” of the syndrome [38]. However, that study did not include a control group of healthy participants.

It is important to note that, when assessing the knowledge of PCOS aspects, participants were given the option to answer with “I am not sure”. Despite that, 23.70% firmly believed that traditional herbs can treat PCOS. This can be explained by the cultural belief in using traditional herbs to treat illnesses in the Middle East, especially gynecological problems such as hormonal imbalances and infertility [39]. Another study reported a prevalence of up to 82.3% use of herbal medicine during pregnancy in the same region [40]. Likewise, 31.90% and 11.80% were confident that good hygiene and vaccines can prevent PCOS, respectively. The fact that PCOS is not caused by an infectious agent reflects the complete lack of knowledge exhibited by participants that chose these options. These irrelevant options were meant to be included in the questionnaire to ensure the accuracy of the knowledge exhibited by participants.

In terms of knowledge sources, participants who chose medical professionals as their source were found to be most aware compared to other participants who chose any other source of information. This reflects the discrepancy in the accuracy of information provided by different sources. However, other findings reflect poor counseling and knowledge exhibited by medical personnel. For instance, more than half of the participants who acquired their knowledge from medical professionals were found to be insufficiently aware of the syndrome. Likewise, over 60% of participants who had a previous diagnosis were not aware enough of their diagnosis, and awareness was demonstrated by only 28.1% of the participants who reported to be studying or working in a medical field.

Though present but inadequate, awareness of PCOS in the population comes from either media sources or diagnosed friends and family members. However, Chiu et al. (2018) found that the online sources of PCOS available to females of the general population are of low quality and lack accreditation and evidence [41], which might explain the inaccurate perceptions of knowledge exhibited by our sample. According to a Canadian study, dissatisfied patients with a rough diagnosis journey of PCOS had to find their own ways of advocacy in an attempt to increase awareness among their friends and family members [42]. However, acquiring knowledge from family and friends can also explain the higher accuracy rates associated with participants’ knowledge about PCOS symptoms.

In this study, the majority of participants reported family and friends as their source of knowledge, while medical professionals and campaigns were least frequently reported. On the other hand, similar studies reported different findings in terms of the most common source of information, reflecting an inconsistency in providing reliable education to the general public [43–47]. This calls for the need not only to educate the general public on the syndrome but also to inform physicians on the importance of thorough patient education and the provision of accurate information. It has been previously exhibited by interviewed patients in the literature that they generally were dissatisfied with their physicians and felt as if “they knew better about the syndrome” than their doctors [48]. The interventional methods chosen to educate the general public have to be well-established as only 5% of the study participants chose campaigns as their source of information—out of which only 14% demonstrated sufficient awareness.

### Limitations

It is important to address that the choice of sampling method was constrained by the fact that data collectors were medical students at the time. A non-probability convenience sampling method was chosen to interview females above the age of 18 residing in Sharjah city, UAE. Thus, this may affect the generalization of the study results. Therefore, there is a need for a similar study to be conducted nationwide for more generalizable results. Moreover, due to the same reason mentioned above, most participants fell in our sample’s 18–24 years old age group. Accordingly, the data obtained can be under-representative of other age groups. Lastly, a small number of participants refused to take part in the interview. As such, none of their responses were recorded, leading to non-response bias. Nonetheless, the correlations concluded from the study findings are still valuable, and can build a scaffolding for future studies.

### Future directions

Promoting PCOS awareness in the population can motivate potential patients to seek medical attention and get an early diagnosis, leading to earlier interventions that are not only cost-efficient but also more effective in improving patients’ quality of life. This can be achieved simply by implementing proper community-wide educational PCOS programs and campaigns [33, 49, 50], in addition to support groups [51], all of which provide accurate and reliable knowledge to the population. These recommendations can be simple yet significant aids to alleviate the burden PCOS has on patients and healthcare.

## Conclusion

In conclusion, there is an evident lack of PCOS awareness among the study participants. The knowledge level that the sample exhibits might reflect an overall lack of knowledge at the population level, which is not sufficient enough to prompt potential patients to seek medical care when necessary, nor it is enough to halt the underlying disease progression. Fortunately, the serious complications caused by PCOS are easily preventable with an early diagnosis, which is only achievable if potential patients are well aware of the syndrome.

## Supplementary Information

**Additional file 1**. English and Arabic PCOS Study Questionnaires

## Data Availability

Data are available upon request from the corresponding author.

## Abbreviations

PCOS: Polycystic Ovarian Syndrome
UAE: United Arab Emirates
SPSS®: Statistical Package for the Social Sciences
MENA: Middle East and North Africa
GCC: Gulf Council Countries
RAK: Ras Al Khaimah
WHO: World Health Organization
