# Supplementary material for: Polycystic ovarian syndrome awareness among females in the UAE: a cross-sectional study
Source: BMC Womens Health. 2023 Apr 17;23:181. doi: 10.1186/s12905-023-02318-y (PMC10108484; doi:10.1186/s12905-023-02318-y)
Supplement: Supplementary file 1 — Additional file 1. English and Arabic PCOS Study Questionnaires [file 12905_2023_2318_MOESM1_ESM.pdf]

University of Sharjah

College of Medicine

Information Sheet for Participants in Research

We are a group of year two medical students at the University Of Sharjah conducting a research project about awareness of Polycystic Ovary Syndrome in the UAE as a requirement in our medical education. The purpose of this study is to describe the level of awareness of Polycystic Ovary Syndrome term, features and complications among females above the age of 18 in the UAE.

You have been selected to participate in this study and your participation is strictly voluntary. If you agree to participate, you will be asked to fill out a questionnaire that will take 6-9 minutes of your time.

There are no risks associated with participation in this study. The questionnaire is anonymous and we assure you that your responses will be confidential and will be used only for the research purposes.

If you have any questions regarding this study or would like to be informed about its results, please feel free to contact Balkis Zaitoun at U17101664@sharjah.ac.ae or Yahya Alassouli at U17100768@sharjah.ac.ae. Filling out this questionnaire indicates your agreement to participate in the study.

For any further concerns, you may contact Dr. Suhail Al Amad, the head of the Research Ethics Committee at University of Sharjah at 06/707304 or our research supervisor Dr. Ghada Mohammed at gmohammed@sharjah.ac.ae.

Please tick the most suitable answer:

1. What is your age in years?
   1. 18-24
   2. 25-34
   3. 35-44
   4. 45-54
   5. 55 and above

2. What is your nationality? …………………….

3. Are you a resident of the UAE?
   1. Yes
   2. No (Go to Question 5)

4. What is your marital status?
   1. Single
   2. Married
   3. Divorced
   4. Widowed

5. What is your educational level?
   1. Did not enter school
   2. Primary School
   3. Middle School
   4. High School Degree / High School Diploma
   5. Diploma / Bachelor's Degree (University)
   6. Higher studies

6. What is your current working status?
   1. Student
   2. Employed/Retired
   3. Unemployed (Go to Question 9)

7. What is your field of work/study?
   1. Medical
   2. Non-medical

8. Have you ever heard of the term PCOS (Polycystic Ovary Syndrome)?
   1. Yes
   2. No

9. Do you know anyone who is diagnosed with PCOS?
   1. Yes
   2. No

10. Have you ever been diagnosed with PCOS?
    1. Yes
    2. No

11. Do you know what are the signs/features of PCOS?
    1. Yes
    2. No

12. Tick all the options that you know are PCOS symptoms?

	1. Yes	2. No	3. I'm not sure
A. Menstrual irregularities			
B. High temperature (fever)			
C. Excessive facial hair			
D. Acne / oily skin			
E. Diarrhea			
F. Weight gain			

13. Do you know what causes PCOS?
    1. Yes
    2. No

14. If yes, tick all what you think causes PCOS:

	1. Yes	2. No	3. I'm not sure
A. Genetics (runs in the family)			
B. Overproduction of hormones			
C. Ageing			
D. Type of food			
E. Infections			
F. Lack of sleep			

15. Can PCOS be prevented?
    1. Yes
    2. No (Go to Question 18)

16. If yes, tick all what you think applies to prevention:

	1. Yes	2. No	3. I'm not sure
A. Healthy exercise			
B. Good diet			
C. Vaccines			
D. Weight control			
E. Good hygiene			
F. Stop smoking			

17. Do you know what are the complications of PCOS?
    1. Yes
    2. No

18. If yes, tick all what you think applies to complications:

	1. Yes	2. No	3. I'm not sure
A. Infertility			
B. Vision problems			
C. Back pain			
D. Uterine cancer			
E. Heart diseases (Cardiovascular problems)			
F. Asthma			

19. Can PCOS be treated?
    1. Yes
    2. No (Go to Question 22)

20. If yes, tick all what you think applies to treatment:

	1. Yes	2. No	3. I'm not sure
A. Healthy diet			
B. Physical Exercise			
C. Surgery			
D. Tablets			
E. Chemotherapy			
F. Traditional herbs			

21. What was your source of information?
    1. Friends or family
    2. Media (Social Media / Internet / TV / etc.)
    3. Medical professionals
    4. Campaigns

Thank you.

جامعة الشارقة

كلية الطب

نموذج للموافقة على الاشتراك كمتطوعة في بحث علمي

نحن مجموعة من طلاب السنة الثانية في كلية الطب في جامعة الشارقة نقوم بإجراء مشروع بحثي عن وعي الإناث في دولة الإمارات العربية المتحدة عن متلازمة تكيس المبايض المتعدد كمتطلب دراسي. الغرض من هذه الدراسة هو وصف مستوى الوعي عن المتلازمة كمصطلح، عن عوارض المتلازمة وعن مضاعفاتها عند إناث الإمارات فوق عمر ال18.

لقد تم اختياركِ عشوائيًا للمشاركة في هذه الدراسة ومشاركتكِ هي أمر اختياري تمامًا. إذا وافقتِ على المشاركة، سوف يُطلب منكِ ملء استبيان لن يأخذ أكثر من 6-9 دقائق من وقتك.
لا توجد مخاطر مرتبطة بالمشاركة في هذه الدراسة. الاستبيان مجهول الهوية ونؤكد لكِ أن إجاباتكِ ستكون سرية ولن تُستخدَم إلا لأهداف البحث العلمي.

إذا كانت لديكِ أي أسئلة بخصوص هذه الدراسة أو ترغبين بأن تكوني على علم بنتائجها، لا تترددي بالتواصل مع **بلقيس زيتون** على U17101664@sharjah.ac.ae أو مع **يحيى العسولي** على U17100768@sharjah.ac.ae.
للمزيد من الاستفسارات، يمكنكِ الاتصال بالدكتور **سهيل العمد**، رئيس لجنة أخلاقيات البحث في جامعة الشارقة على 5057304/06 أو يمكنكِ التواصل مع الدكتور **غادة محمد**، الدكتورة المشرفة على بحثنا، على gmohammed@sharjah.ac.ae.

يشير ملء هذا الاستبيان إلى موافقتكِ على المشاركة في الدراسة.

1) ما هو عمركِ بالسنوات؟
   1. 18-24
   2. 25-34
   3. 35-44
   4. 45-54
   5. >55

2) ما هي جنسيتكِ؟ ____________________

3) هل أنتِ مقيمة في دولة الإمارات العربية المتحدة؟
   1. نعم
   2. لا

4) ما هي حالتكِ الاجتماعية؟
   1. غير مرتبطة
   2. متزوجة
   3. مطلقة
   4. أرملة

5) ما هو مستواكِ التعليمي؟
   1. لم أدخل المدرسة
   2. المدرسة الابتدائية
   3. المدرسة المتوسطة
   4. شهادة / دبلوم الثانوية العامة
   5. دبلوم / درجة البكالوريوس في الجامعة
   6. دراسات عليا

6) ما هو وضع عملكِ الحالي؟
   1. طالبة
   2. صاحبة عمل / متقاعدة
   3. عاطلة عن العمل (انتقلي إلى سؤال 8)

7) ما هو مجال عملكِ / دراستكِ؟
   1. مجال طبي
   2. مجال غير طبي

8) هل سمعتِ من قبل عن مصطلح متلازمة تكيس المبايض المتعدد؟
   1. نعم
   2. لا

9) هل تعرفين أي شخص مصاب بمرض متلازمة تكيس المبايض المتعدد؟
1. نعم
2. لا

10) هل سبق وتم تشخيصك بمتلازمة تكيس المبايض المتعدد؟
1. نعم
2. لا

11) هل تعلمين ما هي علامات وأعراض متلازمة تكيس المبايض المتعدد؟
1. نعم
2. لا (انتقلي إلى سؤال 13)

12) ضعي علامة صح بجانب جميع الخيارات التي تعتقدين أنها تنطبق على أعراض متلازمة تكيس المبايض المتعدد.

	1. نعم	2. لا	3. لستُ متأكدة
عدم انتظام الدورة الشهرية			
ارتفاع درجة حرارة الجسم			
شعر الوجه الزائد			
حب الشباب / البشرة الدهنية			
الإسهال			
زيادة الوزن			

13) هل تعلمين ما أسباب الإصابة بمتلازمة تكيس المبايض المتعدد؟
1. نعم
2. لا (انتقلي إلى سؤال 15)

14) إذا كانت إجابتكِ نعم في السؤال السابق، ضعي علامة صح بجانب جميع الخيارات التي تعتقدين أنها تسبب متلازمة تكيس المبايض المتعدد.

	1. نعم	2. لا	3. لستُ متأكدة
وراثي (عبر العائلات)			
الإفراز المفرط للهرمونات في الجسم			
كبر السن			
نوع الأطعمة			
الإلتهابات			
قلة النوم			

15) هل تعتقدين أنه من الممكن الوقاية من متلازمة تكيس المبايض المتعدد؟

1. نعم
2. لا (انتقلي إلى سؤال 17)

16) إذا كانت إجابتكِ نعم في السؤال السابق، ضعي علامة صح بجانب جميع الخيارات التي تعتقدين أنها قد تقي من متلازمة تكيس المبايض المتعدد.

	1. نعم	2. لا	3. لستُ متأكدة
التمارين الصحية			
حمية غذائية جيدة			
التطعيمات			
ضبط الوزن			
النظافة			
الإقلاع عن التدخين			

17) هل تعلمين ما هي مضاعفات الإصابة بمتلازمة تكيس المبايض المتعدد؟
1. نعم
2. لا (انتقلي إلى سؤال 19)

18) إذا كانت إجابتكِ نعم في السؤال السابق، ضعي علامة صح بجانب جميع الخيارات التي تعتقدين أنها قد تكون نتيجة الإصابة بمتلازمة تكيس المبايض المتعدد.

	1. نعم	2. لا	3. لست متأكدة
العقم			
مشاكل في النظر			
ألم في الظهر			
سرطان الرحم			
الأمراض القلبية. (أمراض الجهاز الدموي)			
الربو			

19) هل تعتقدين أن متلازمة تكيس المبايض يمكن علاجها؟
1. نعم
2. لا (انتقلي إلى سؤال 21)

20) إذا كانت إجابتكِ نعم في السؤال السابق، ضعي علامة صح بجانب جميع الخيارات التي تعتقدين أنها قد تساهم في علاج متلازمة تكيس المبايض المتعدد.

	1. نعم	2. لا	3. لست متأكدة
الأكل الصحي (الحمية الصحية)			
التمارين الرياضية			
العمليات الجراحية			
تناول الحبوب (الأدوية)			
العلاج الكيميائي			
العلاج التقليدي			

21) ما هو مصدر معلوماتك؟

1. الأقارب أو الأصدقاء
2. الإعلام (وسائل التواصل الإجتماعي، الإنترنت، التلفاز، الخ...)
3. ممارس طبي
4. الحملات التوعوية

شكراً جزيلاً لكِ.
